# Complete mitogenome and phylogenetic analysis of hide beetle *Dermestes maculatus* (Insecta, Coleoptera, Dermestidae)

**DOI:** 10.1080/23802359.2017.1407708

**Published:** 2017-11-24

**Authors:** Mustafa Zafer Karagozlu, Seong Hwan Park, Sang-Eon Shin, Chang-Bae Kim

**Affiliations:** a Department of Biotechnology, Sangmyung University, Seoul, Korea;; b Department of Legal Medicine, Korea University College of Medicine, Seoul, Korea

**Keywords:** Insecta, coleoptera, dermestidae, complete mitogenome, *Dermestes maculatus*

## Abstract

In this study, the complete mitochondrial genome of hide beetle *Dermestes maculatus* which was collected from Seoul, South Korea was sequenced by next-generation sequencing. The size of mitochondrial genome is 17,026 bp that composed of 13 protein coding, two ribosomal RNA and 22 tRNA genes which has the identical gene orientation with the other Bostrichiformia species. Additionally, the phylogenetic tree of the *D. maculatus* in the infraorder Bostrichiformia was reconstructed by using 13 protein-coding genes of complete mitochondrial genome. The results showed that the family Dermestidae is positioned in the infraorder Bostrichiformia early branched than family Bostrichidae. This study provides the first complete mitochondrial genome from the genus *Dermestes.*

*Dermestes maculatus* (hide beetle) is a black hairy beetle species which belongs to the family Dermestediae. They have ability to eat carcase until remain skeleton (Hoermann et al. 2011). This habit makes them significantly important for forensic investigations to estimating the post mortem interval in suicide or homicide cases (Magni et al. 2015). The adult *D. maculatus* specimens generally arrive within 5–11 days following death (Richardson and Goff 2001) and they stay until late stages. Even their larvae can collected during the dry stage and remains stage (days 25–66+) (Richards and Goff 1997). In spite of the forensic importance of the species, there is no record for the complete mitochondrial genome of the Dermestidae species. In the present study, we are providing the complete mitochondrial genome of the *D. maculatus.* This is the first complete mitgenome record from the genus.

The *D. maculatus* specimen were collected from forest in Gangdong-gu, Seoul, Korea 37°32′38″ N, 127°09′25″ E and identified by DNA barcoding (Park and Shin 2013). The specimens deposited in Department of Legal Medicine, Korea University (16Ma20). After extracting of total DNA from the legs and thorax, the complete mitogenome sequenced and phylogenetic relationships investigated. The methods for analysis explained in our previous article (Karagozlu et al. 2016).

The size of the mitogenome is 16,390 bp that composed of 13 protein coding, 2 ribosomal RNA and 22 tRNA genes (GenBank accession no. MG457037). This is the longest mitogenome among records which belong to the family Bostrichidae. Rearrangements of gene positions and structures are typical in insect mitogenomes (Cameron 2014). Likewise, all dipteran records 23 genes encoded on the majority strand and 14 genes positioned in minority strand. The nucleotide composition of the genome is 40.3% A, 13.4% C, 10.2% G, and 36.1% T. Total A–T content is 78.5% for complete mitochondrial genome and it is 71.4% for 13 protein-coding genes. The entire non-coding A + T rich region area between12S rRNA and tRNA-Ile is typically annotated as the ‘control region’ or the ‘in the insect mitogenome (Cameron 2014). In the mitogenome of the *D. maculatus,* the length of this region is 1263 bp.

Furthermore, the molecular phylogeny of the *D. maculatus* investigated in the infraorder Bostrichiformia. For phylogenetic analysis, 13 protein-coding genes of complete mitochondrial genome were conducted. The results showed that the family Dermestidae is positioned in the infraorder Bostrichiformia and earlier branched than the family Bostrichidae which is represented with a clade that include three species (Figure 1). Unfortunately the complete mitochondrial genome records in the GenBank were limited to confirm exact position of the species in the phylogenetic tree. Nevertheless, the previous study based on nuclear 18S rRNA, and mitochondrial 16S rRNA and COX1 genes also showed the similar results (Hunt et al. 2007). Besides, the results of the study used nuclear 28S rRNA, and mitochondrial 16S rRNA and COX1 is also similar (Bell and Philips 2012). This study provides genomic data for mitochondrial genome library to investigate evolutionary and systematic studies of the family Dermestidae.

**Figure 1.**
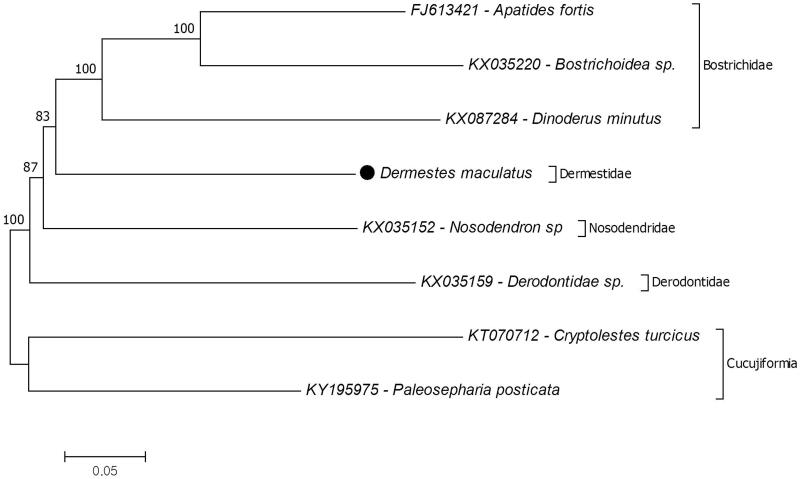
Molecular phylogeny of the *D. maculatus* in the infraorder Bostrichiformia. The phylogenetic tree was reconstructed by using protein-coding genes of complete mitochondria. The outgroup species *Cryptolestes turcicus* and *Paleosepharia posticata* were chosen from the infraorder Cucujiformia. The complete mitochondrial genome data retrieved from GenBank.
